# Galactomannan-Decorated Lipidic Nanocarrier for Gene Supplementation Therapy in Fabry Disease

**DOI:** 10.3390/nano12142339

**Published:** 2022-07-08

**Authors:** Julen Rodríguez-Castejón, Itziar Gómez-Aguado, Marina Beraza-Millor, María Ángeles Solinís, Ana del Pozo-Rodríguez, Alicia Rodríguez-Gascón

**Affiliations:** 1 Pharmacokinetic, Nanotechnology and Gene Therapy Group (PharmaNanoGene), Faculty of Pharmacy, Centro de Investigación Lascaray Ikergunea, University of the Basque Country (UPV/EHU), Paseo de la Universidad 7, 01006 Vitoria-Gasteiz, Spain; julen.rodriguez@ehu.eus (J.R.-C.); itziar.gomez@ehu.eus (I.G.-A.); marina.beraza@ehu.eus (M.B.-M.); marian.solinis@ehu.eus (M.Á.S.); 2 Bioaraba, Microbiology, Infectious Disease, Antimicrobial Agents, and Gene Therapy, 01006 Vitoria-Gasteiz, Spain

**Keywords:** advanced therapies, Fabry disease, galactomannan, gene augmentation, intravenous administration, non-viral vectors, plasmid DNA, solid lipid nanoparticles, targeting, α-galactosidase A

## Abstract

Gene supplementation therapy with plasmid DNA (pDNA) represents one of the most promising strategies for the treatment of monogenic diseases such as Fabry disease (FD). In the present work, we developed a solid lipid nanoparticles (SLN)-based non-viral vector with a size below 100 nm, and decorated with galactomannan (GM) to target the liver as an α-Galactosidase A (α-Gal A) production factory. After the physicochemical characterization of the GM-SLN vector, cellular uptake, transfection efficacy and capacity to increase α-Gal A activity were evaluated in vitro in a liver cell line (Hep G2) and in vivo in an animal model of FD. The vector showed efficient internalization and it was highly efficient in promoting protein synthesis in Hep G2 cells. Additionally, the vector did not show relevant agglutination of erythrocytes and lacked hemolytic activity. After the systemic administration to Fabry mice, it achieved clinically relevant α-Gal A activity levels in plasma, liver, and other organs, importantly in heart and kidneys, two of the most damaged organs in FD. This work shows the potential application of GM-decorated lipidic nanocarries for the treatment of FD by pDNA-based gene augmentation.

## 1. Introduction

Fabry disease (FD) is a lysosomal storage disorder (LSD) caused by mutations in the gene *GLA* in the X chromosome, which is responsible for encoding the lysosomal enzyme α-Galactosidase A (α-Gal A). As a result, glycosphingolipids (mainly globotriaosylceramide (Gb3) and its deacylated derivative globotriaosylsphingosine (lyso-Gb3)) are progressively accumulated in the lysosome of the cells, especially in vascular endothelial and smooth muscle cells, leading to cardiac, renal and cerebrovascular manifestations [1]. Currently, two treatment strategies are available for the treatment of FD: intravenous (i.v.) enzyme replacement therapy (ERT) with recombinant enzymes agalsidase α (Replagal^®^) or agalsidase β (Fabrazyme^®^), and an oral chaperone, Migalastat^®^. Although existing therapies have been shown to improve the quality of life of patients, none have been able to completely revert clinical manifestations and there are still many clinical needs to be met [2,3]. For instance, ERT shows considerable clinical variation and the effect is determined by the initiation age [4], and chaperone therapy is only effective in patients with certain mutant forms of α-Gal A that are amenable to the treatment [5].

The application of gene augmentation therapy for the treatment of FD represents a promising future for the management of this rare genetic disease. Delivery of the *GLA* sequence by plasmid DNA (pDNA) or messenger RNA (mRNA) administration to express α-Gal A in native cells has been proposed to overcome the limitations of current therapies [6,7]. Endogenously produced α-Gal A by gene supplementation is advantageous because it involves natural translational and post-translational modifications, enhancing stability and reducing immunogenicity as compared to recombinant α-Gal A [8,9,10,11,12]. Studies in animal models of Fabry disease administered with mRNA have shown promising results [13,14], but gene augmentation with pDNA is expected to provide a long-lasting gene expression and purified pDNA can be obtained more easily and inexpensively than mRNA [15]. 

Achieving a relevant α-Gal A activity in the most damaged organs represents a major challenge for gene therapy. α-Gal A is secreted into the systemic circulation to be recaptured by neighboring and non-neighboring cells. This cross-correction phenomenon allows certain organs to act as an enzyme factory, where the α-Gal A is produced and released into the blood circulation to reach the rest of the organs [16]. Different authors have proposed the liver as a target organ for the treatment of FD by gene augmentation therapy [13,14,17,18,19,20,21]. Hepatic cells can be transfected in vivo with the *GLA* sequence, and expressed α-Gal A can leave the liver and reach distant affected organs via systemic circulation [22]. 

Systemic gene therapy implies the use of an appropriate delivery system that protects the genetic material after administration, carries it to the target organ and releases it into the cell of interest providing an adequate intracellular disposition [23]. 

Lipid nanoparticles (LNs) are currently the leading technology enabling non-viral gene therapy. LNs have been of great interest for many years due to their safe profile, and in recent years, their clinical evaluation for gene therapy has increased dramatically. The need for the rapid development and large-scale production of an effective technology to mitigate the consequences of the coronavirus disease 2019 (COVID-19) pandemic marked a milestone in the field of nanotechnology [24,25,26]. The approval of two lipid-based mRNA vaccines against SARS-CoV-2 has placed LNs at the forefront of non-viral platforms for nucleic acid delivery. 

LNs are very versatile delivery platforms composed of biocompatible and biodegradable excipients, generally approved for pharmaceutical use, and produced by easy and scalable methods [27]. Additionally, LNs can be decorated with ligands on the surface to control biodistribution and improve selectivity to a certain tissue or type of cells [28]. In previous works, we had developed and evaluated in vitro and in vivo a multicomponent non-viral vector based on a type of LNs (solid lipid nanoparticles, SLNs) decorated with the polysaccharide dextran [29,30,31,32], as delivery systems of pDNA. These SLN-based vectors increased α-Gal A activity in different organs after i.v. administration to FD model mice [32], but the need to improve the selectivity of the system for certain organs, such as the liver, was noticed. In this regard, the size of the carrier plays an important role in the in vivo delivery of actives to hepatocytes. To reach parenchymal liver cells, it is necessary to overcome the sinusoidal fenestrate, which has a size between 100 nm and 200 nm, depending on the species [33]. Additionally, liver specificity can be improved by functionalizing nanocarriers with moieties with high tissue-affinity, such as galactosyl ligands, which are efficiently taken up by liver cells [34]. 

With this aim in mind, a new SLN-based vector has been designed to address FD by gene augmentation therapy targeted to the liver. In this work, we evaluated the capacity of the new SLN-based vector with the pDNA encoding α-Gal A to increase in vivo enzyme activity levels in plasma and tissues after i.v. administration to FD mice. Moreover, the influence of particle size and functionalization with galactomannan (GM) polysaccharide on the biodistribution of in situ expressed α-Gal A was studied. 

The SLN-based vector functionalized with GM was around 100 nm in size and showed a highly efficient internalization and protein production capacity in vitro in a liver cell model. The GM-SLN vector presented suitable characteristics and compatibility for i.v. administration and produced clinically relevant levels of α-Gal A activity in plasma and tissues. The development of this new GM-SLN vector offers a new approach to the management of FD.

## 2. Materials and Methods

### 2.1. Materials

1,2-Dioleoyl-3-trimethylammonium-propane chloride salt (DOTAP) was acquired from Avanti Polar-lipids, Inc. (Alabaster, AL, USA). Precirol^®^ ATO 5 (glyceryl palmitostearate) was generously provided by Gattefossé (Madrid, Spain). Tween 80 was purchased from Panreac (Madrid, Spain). Sigma-Aldrich (Madrid, Spain) provided protamine sulfate salt from salmon (Grade X) (P), D-Galacto-D-mannan from *Ceratonia siliqua* (Mr ~200,000) (GM) and Nile Red.

Plasmid pcDNA3-EGFP (6.1 kb) encoding the green fluorescent protein (GFP) was kindly provided by the laboratory of Professor B.H.F. Weber (University of Regensburg, Regensburg, Germany). Plasmid pR-M10-αGal A was purchased from Origene (Rockville, MD, USA).

Materials used for agarose gel electrophoresis assay were acquired from Bio-Rad (Madrid, Spain). Deoxyribonuclease I (DNase I) and sodium dodecyl sulfate (SDS) were purchased from Sigma-Aldrich and GelRed™ from Biotium (Fremont, CA, USA).

Human hepatocellular carcinoma cells (Hep G2 [HEPG2] (ATCC HB-8065)) and Eagle’s Minimum Essential Medium (EMEM) were purchased from American Type Culture Collection (ATCC, Manassas, VA, USA). Cell culture reagents, including, fetal bovine serum (FBS), Penicillin-Streptomycin, Trypsin/EDTA, HEPES buffered solution and Phosphate-Buffered Saline (PBS) were acquired from Gibco (Thermo Fisher Scientific, Madrid, Spain). Lipofectamine^®^ 2000 Lipid-Reagent was purchased from Invitrogen (Thermo Fisher Scientific, Madrid, Spain), 4′,6-diamidine-2′-phenylindole dihydrochloride (DAPI)-fluoromount-G^®^ from Southern Biotech (Birmingham, AL, USA) and the 7-Amino-Actinomycin D (7-AAD) Viability Dye from Beckman Coulter (Brea, CA, USA).

4-methylumbelliferyl-α-D-galactopyranoside (4-MU-α-Gal), N-acetyl-D-galactosamine and 4-methylumbelliferone (4-MU) were obtained from Sigma-Aldrich (Madrid, Spain). Micro BCA™ Protein Assay Kit was acquired from Thermo Fisher Scientific (Madrid, Spain).

α-Gal A knockout (KO) mice (B6;129-Glatm1Kul/JAX stock #003535) [35] were purchased from The Jackson Laboratory (Bar Harbor, ME, USA).

Other chemicals, if not specified, were reagent grade chemicals from Sigma-Aldrich (Madrid, Spain) and Panreac (Barcelona, Spain).

### 2.2. Preparation of SLNs

SLNs were prepared by a hot-melt emulsification technique. The preparation process of SLNs was optimized to obtain nanoparticles around 100 nm in size. To this end, DOTAP (0.4% *w/v*) and Tween 80 (0.1% *w/v*) were dissolved in Milli-Q™ water (EDM Millipore, Billerica, MA, USA) to obtain the aqueous phase. The oil phase consisted of Precirol^®^ ATO 5. Both phases were heated to 80 °C in a bain-marie and the aqueous phase was added to the oily phase and immediately sonicated (Branson Sonifier 250, Danbury, CT, USA) at 50 W. Then, the emulsion was subjected to a cold-shock in an ice bath to obtain a suspension of SLNs upon solidification of the Precirol^®^ ATO 5 in the aqueous medium. Two different factors were evaluated to optimize the process: amount of Precirol^®^ ATO 5 (100 or 200 mg), and sonication time (3, 5, 7.5, 10, 15, 20 and 30 min). The amount of 100 mg of Precirol^®^ ATO 5 was chosen in accordance with our previous studies with other SLN formulations prepared by a different technique [29,31,32,36]. We also evaluated here the influence on the properties of nanoparticles prepared by hot-melt emulsification with a higher solid lipid content, 200 mg of Precirol^®^ ATO 5. 

### 2.3. Physical Stability of SLNs

The stability of the SLN suspension at 4 °C was assessed for five weeks. Every week, particle size, polydispersity index, and ζ-potential were measured, as described in Section 2.5.

### 2.4. Formulation of the SLNs-Based Vector with Galactomannan (GM-SLN)

To obtain the GM-SLN nanovector, a solution of protamine (P) was first mixed with a pDNA that encodes a reporter green fluorescent protein (GFP) or α-Galactosidase A (α-Gal A) (pcDNA3-EGFP or pR-M10-αGal A, respectively) for 5 min to form a P-pDNA complex at *w/w* ratio 2:1. Then, a solution of galactomannan (GM) was incorporated at GM-P-pDNA ratios of 0.1:2:1 (*w/w/w*) and mixed for 15 min. Finally, SLNs were added and incubated for 20 min at room temperature. Interactions between the components led to the adsorption of the GM-P-pDNA complexes on the surface of the SLNs, resulting in the formation of the final GM-P-pDNA-SLN vector at *w/w/w/w* ratio of 0.1:2:1:2. The vector was prepared 24 h prior to use and stored at 4 °C.

For in vivo experiments, the vector was concentrated to 0.4 µg/µL of pDNA by vacuum centrifugation (2000 rpm, 40 °C, 16 mbar) in a HyperVAC™ Centrifugal Vacuum Concentrator (Gyrozen^®^, Gimpo, Korea). 

### 2.5. Characterization of SLNs and the GM-SLN Vector: Particle Size, Polydispersity Index and ζ-Potential Measurements

Dynamic Light Scattering (DLS) was employed to determine particle size and polydispersity index, and Laser Doppler Velocimetry (LDV) to measure ζ-potential. Samples were diluted in Milli-Q™ water (EDM Millipore, Billerica, MA, USA) and measurements were carried out in a ZetaSizer Nano ZS (Malvern Instruments, Worcestershire, UK).

### 2.6. Fourier Transform Infrared Spectroscopy (FT-IR) of SLNs and the GM-SLN Vector

For the determination of interactions between the components of the GM-SLN vector, Fourier Transform Infrared Spectroscopy (FT-IR) analysis was performed. SLNs and the GM-SLN vector were lyophilized at −50 °C and 0.2 mbar for 42 h (LyoBeta 15, Telstar, Spain) and the powders were mixed homogeneously with potassium bromide. The blends were compressed using a hydraulic compressor applying a pressure of 10 tons for 5 min. The discs obtained were placed in an FT-IR spectrometer (Thermo Fisher Scientific, Madrid, Spain) and the IR spectrums were recorded in the mid-IR region (4000–400 cm^−1^). The recorded signals were reported as transmittance percentages.

### 2.7. Cryo-Transmission Electron Microscopy (Cryo-TEM) Images

The morphology of the SLNs and the GM-SLN vector was analyzed by transmission electron microscopy (TEM), in a TECNAI G2 20 TWIN (FEI), operating at an accelerating voltage of 200 KeV in a bright-field image mode and low-dose image mode. A 3 µL aliquot of sample solution was placed on glow-discharged 300 mesh Quantifoil TEM grids and used for plunge freezing into liquid ethane on an FEI Vitrobot Mark IV (Eindhoven, The Netherlands). The frozen grids were then transferred to a 626 DH Single Tilt Cryo-Holder (Gatan, France), where temperature was maintained below −170 °C (liquid nitrogen temperature) and then were transferred to TEM at liquid nitrogen temperature.

### 2.8. Binding, Protection and Release of the pDNA

The capacity of the vector to bind the pDNA (pcDNA3-EGFP or pR-M10-αGal A), to protect it against DNase I digestion and to release it was studied by 0.7% (*w/v*) agarose gel electrophoresis labeled with GelRed™. The gel was run for 30 min at 120 V and analyzed with an Uvitec Uvidoc D-55-LCD-20 M Auto transilluminator (Cambridge, UK). The binding capacity was evaluated by adding directly in the gel the vector diluted in Milli-Q™ water to a final concentration of 0.03 µg pDNA/µL. To study the protection against DNase I digestion, the vector at the same concentration was incubated at 37 °C for 30 min in the presence of 1.5 U DNase I/2.5 µg pDNA. Then, a solution of SDS (4% *w/v*) was added to a final concentration of 1% (*w/v*) and incubated at room temperature. In the release study, the same SDS solution was added to the vector to unbind the pDNA. Two controls for the integrity of the pDNA were included in the gel: 1 Kb pDNA ladder from Thermo Fisher Scientific (Madrid, Spain) and untreated pcDNA3-EGFP or pR-M10-αGal A. 

### 2.9. In Vitro Studies in Hep G2 Cells

#### 2.9.1. Cell Culture and Transfection Protocol

Human hepatocellular carcinoma (Hep G2) cells were cultured in Eagle’s Minimum Essential Medium (EMEM), supplemented with 10% (*v/v*) heat-inactivated fetal bovine serum (FBS) and 1% (*v/v*) penicillin-streptomycin. Cells were incubated at 37 °C in a 5% CO_2_/95% air atmosphere and passaged every 2–3 days at 70–90% confluence. For all experiments, cells were used at passage 4–8 post-thaw.

For in vitro assays, cells were seeded on 24-well plates at a density of 120,000 cells/well and allowed to adhere overnight. The GM-SLN vector was diluted in HBS and 75 µL of the vector carrying 2.5 µg of pDNA (pcDNA3-EGFP or pR-M10-αGal A) were added to each well, and incubated at 37 °C in a 5% CO_2_/95% air atmosphere for 2 h (cellular uptake and intracellular disposition assays) or 4 h (transfection assays). After the corresponding incubation times, the medium containing the vector was removed and cells were supplemented with fresh complete medium before proceeding with downstream experiments. Lipofectamine^®^ 2000 was employed as positive control of transfection in accordance with the manufacturer’s instructions.

#### 2.9.2. Cellular Uptake

The internalization of the GM-SLN vector into the cells was studied by flow cytometry using SLNs labeled with the fluorescent dye Nile Red (λ = 590 nm), as previously described [36]. Two hours after the addition of the Nile Red-labeled GM-SLN vector, cells were washed with phosphate-buffered saline (PBS) and detached from the plates with 0.05% trypsin/EDTA. The cell suspensions were centrifuged at 1000 rpm for 5 min, and pellets were resuspended in PBS for flow cytometry analysis. The cellular uptake of the vector was analyzed using a CytoFLEX flow cytometer (Beckman Coulter) at 610 nm (ECD). The percentage of positive cells corresponds to the cells that have internalized the vector with Nile Red-labeled SLNs over the total cells. For each sample, 10,000 events were collected.

#### 2.9.3. Intracellular Disposition

To evaluate the intracellular location of the GM-SLN vector after the internalization into the cells, 120,000 cells in 1 mL of culture medium were seeded in Millicell EZ slides (Millipore) 24 h before the experiment. Nile Red-labeled GM-SLN vectors were added to cultured cells and after 2 h of incubation, the slides were washed with PBS, fixed with 4% paraformaldehyde and covered with the mounting fluid DAPI-fluoromount-G^®^, used to label the nuclei. The slides were then observed under a Leica DM IL LED Fluo inverted microscope (Leica Microsystems CMS GmbH, Wetzlar, Germany).

#### 2.9.4. GFP Transfection Efficacy and Cell Viability

Transfection efficacy of the GM-SLN vector bearing pcDNA3-EGFP, and cell viability were assessed by flow cytometry 3 days post-transfection. Cells were detached from the plates as described in Section 2.9.2, and analyzed in a CytoFLEX flow cytometer (Beckman Coulter). For each sample, 10,000 events were collected.

Transfection efficacy was measured at 525 nm (FITC) [37], and was analyzed in terms of the percentage of transfected cells and the mean intensity of fluorescence. The percentage of transfected cells was quantified by counting GFP-positive cells over total cells. The intensity of fluorescence represented the mean of the intensity of fluorescence per labeled cell, which is correlated with gene expression and protein production [38,39].

To evaluate cell viability, samples were treated with 7-Amino-Actinomycin D (7-AAD) viability dye and viable cells were determined by flow cytometry at 610 nm (ECD).

#### 2.9.5. α-Galactosidase A Transfection

To evaluate the expression of α-Gal A after the addition of the GM-SLN vector bearing pR-M10-αGal A, 3 and 5 days post-transfection culture mediums of cells were collected, and a fluorometric assay was conducted to determine the α-Gal A activity. Culture mediums were centrifuged at 1500 rpm for 10 min at 4 °C, and enzyme activity was quantified as described in Section 2.12. 

### 2.10. Interaction with Erythrocytes: Hemolysis and Hemagglutination 

Hemolytic and hemagglutination effects of the GM-SLN vector bearing pR-M10-αGal A were assessed based on the protocol described by Kurosaki et al. [40]. Fresh human blood was centrifuged at 4000 rpm for 5 min and the plasma and the buffy coat were discarded. Erythrocytes were washed three times with PBS by centrifugation at 4000 rpm for 5 min. Then, they were diluted in PBS to a final concentration of 2% (*v/v*) and 5% (*v/v*) for agglutination and hemolysis studies, respectively. The GM-SLN vector was added to erythrocytes suspension at ratio 1:12 (*v/v*) and incubated at room temperature for 60 min for the hemolysis assay, and 15 min for the hemagglutination assay. After incubation, the samples from the hemolysis assay were centrifuged at 4000 rpm during 5 min and the hemolysis was quantified by measuring hemoglobin release in the supernatant at a wavelength of 545 nm using a Glomax^®^-Multi Detection System (Promega, Madison, WI, USA) microplate reader. A lysis buffer was used as the 100% hemolysis sample. Fifteen µL of the agglutination sample was placed on a microscope slide and observed by an optic inverted microscope (Nikon TMS, Izasa Scientific, Madrid, Spain) at 20× and 40× magnifications. As a positive control of agglutination, a vector prepared at 1:10 (*w/w*) pDNA:Poly-L-Lysine was employed.

### 2.11. Animal Experimentation

All animal experiments were approved by the Ethics Committee on Animal Experimentation (CEEA) of the University of the Basque Country (UPV/EHU) and by the Servicio de Ganadería of the Diputación Foral de Álava (approved protocol M20/2017/157) following the Spanish and European Union (EU) laws, and all the procedures were performed in accordance. 

For in vivo experiments, α-Gal A KO mice (B6;129-Glatm1Kul/JAX stock #003535) were used as FD model animals [35]. Breeding pairs were mated according to mating recommendations from The Jackson Laboratory and their offspring were genotyped by the investigation general services (SGIker) from the University of the Basque Country (UPV/EHU) as described in the genotyping protocol from the Jackson Laboratory website. Animals were housed under controlled temperature, humidity and 12 h light/dark cycles, and had ad libitum access to standard rodent chow and water.

#### 2.11.1. In Vivo Intravenous Administration to α-Gal A KO Mice

Six α-Gal A KO male mice (8–9 weeks old) weighing between 20 and 25 g were divided into two experimental groups (n = 3 per group; untreated and treated). Animals corresponding to the treated group were anesthetized by 1–2% isoflunare (IsoFlo, Abbott, Madrid, Spain) inhalation in air, at a flow rate of 0.5–1 L/min, and intravenously administered with 150 µL of the GM-SLN vector (60 µg of pR-M10-αGal A). At day 5 mice from untreated and treated groups were humanely sacrificed by cervical dislocation. Blood was collected by cardiac puncture on euthanized animals, centrifuged at 5000 rpm for 8 min 4 °C, and obtained plasma was stored at −80 °C until its analysis, as described in Section 2.12. 

Liver, spleen, heart and kidney were harvested from each mouse and stored at −80 °C for analysis. Tissues were homogenized by an MT-3K mini handheld homogenizer (Hangzhou Miu Instruments Co., Ltd., Hangzhou, China) and centrifuged at 12,000× *g* at 4 °C for 10 min. Supernatants were collected for the α-Gal A activity assay and proceeded as described in Section 2.12. 

### 2.12. α-Galactosidase A Activity Assay

α-Gal A activity was measured by a fluorometric assay based on the conversion of 4-methylumbelliferyl-α-D-galactopyranoside (4-MU-α-Gal) into the product 4-methylumbelliferone (4-MU). 

An aliquot of each sample was incubated with 4-MU-α-Gal (5 mM) and a specific inhibitor of α-N-acetylgalactosaminidase (α-Galactosidase B), N-acetyl-D-galactosamine (100 mM), in 0.1 M sodium citrate buffer (pH = 4.4) at 37 °C under agitation. The reaction was stopped with 0.1 M glycine-NaOH buffer (pH = 10.4). The resultant product 4-MU was determined by measurement of fluorescence (λexcitation = 360 nm; λemission = 450 nm) on a Glomax^®^-Multi Detection System (Promega, Madison, WI, USA). Micro BCA™ protein assay was performed to determine protein concentrations. One unit of α-Gal A activity is equivalent to the hydrolysis of 1 nmol of the substrate 4-MU-α-Gal in 1 h at 37 °C. α-Gal A activity was expressed as 4-MU nmol/h/mg total protein or 4-MU nmol/h/mL culture medium/plasma.

### 2.13. Data Analysis and Statistics

Results are expressed as the mean ± standard deviation. All statistical computations were performed using IBM SPSS Statistics 26 (IBM Corp, Armonk, NY, USA). Normal distribution of data was evaluated by the Shapiro–Wilk test and homogeneity of variances by Levene’s test. To compare means of two independent groups, the parametric Student’s *t*-test was employed. For multiple comparisons one-way ANOVA was performed. If variances were homogeneous, the Bonferroni post-hoc test was used. If variances were not homogeneous, the Tamhane post-hoc test was performed. *p* < 0.05 was considered statistically significant in all analyses. 

## 3. Results

### 3.1. Preparation of SLNs

**Figure 1:**
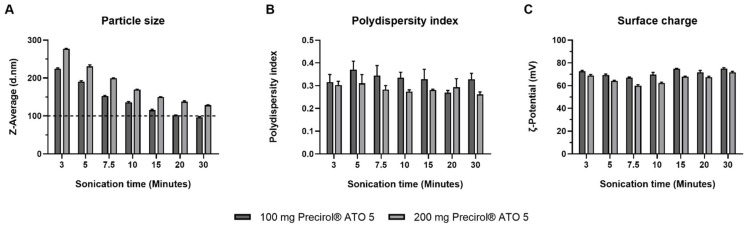
Optimization of the preparation process of SLNs. (**A**) Particle size, (**B**) polydispersity index and (**C**) surface charge of SLNs prepared with 100 or 200 mg of Precirol^®^ ATO 5 at different sonication times. Results are shown as the mean ± standard deviation (*n* = 3). Horizontal discontinuous line refers to a particle size of 100 nm.

The preparation process of the SLNs was optimized in terms of amount of Precirol^®^ ATO 5 and sonication time. Figure 1 features the influence of sonication time on the particle size (A), polydispersity index (B) and ζ-potential (C) of SLNs formulated with 100 or 200 mg of Precirol^®^ ATO 5.

As Figure 1 shows, increasing the sonication time reduced particle size of SLNs, regardless of the amount of Precirol^®^ ATO 5. After 30 min sonication, SLNs with 100 or 200 mg of Precirol^®^ ATO 5 had a particle size of 96.4 and 128.1 nm, respectively. All the formulations showed polydispersity index under 0.4 and positive surface charge. 

The parameters for the synthesis of SLNs for the following experiments were set at 100 mg of Precirol^®^ ATO 5 and 30 min sonication, since the particle size was below 100 nm (95.0 ± 0.9 nm), with a polydispersity index of 0.27 ± 0.01 and and a ζ-potential of +72.3 ± 3.3 mV. 

### 3.2. Physical Stability of SLNs

**Figure 2:**
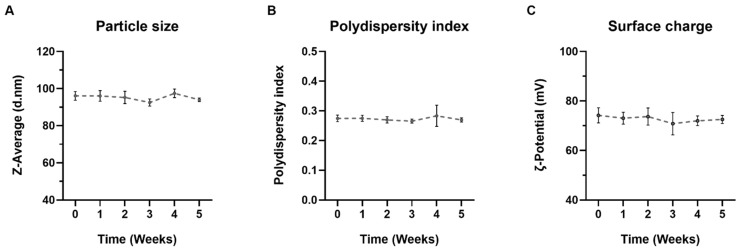
Five-week stability of SLNs at 4 °C. (**A**) Particle size, (**B**) polydispersity index and (**C**) surface charge of SLNs during five weeks stored at 4 °C. Results are shown as the mean ± standard deviation (*n* = 3).

The stability of the SLNs at 4 °C was tested once a week, during five weeks. Figure 2 represents the particle size (A), polydispersity index (B) and ζ-potential (C) of SLNs during the study period. SLNs were stable and no changes in particle size, polydispersity index and ζ-potential were observed.

### 3.3. Characterization of the GM-SLN Vector: Particle Size, Polydispersity Index and ζ-Potential Measurements

Table 1 shows the particle size, polydispersity index and ζ-potential of the GM-SLN vector containing either pcDNA3-EGFP or pR-M10-αGal A. The addition of the pDNA, the P and the GM did not affect particle size, but significantly decreased surface charge to +33.6 ± 1.6 mV. The type of plasmid did not affect the particle size, polydispersity index and ζ-potential of the vector. No significant differences in particle size, polydispersity index and ζ-potential were observed when SLNs were labeled with Nile Red (data not shown). 

### 3.4. Fourier Transform Infrared Spectroscopy (FT-IR) of SLNs and the GM-SLN Vector

**Figure 3:**
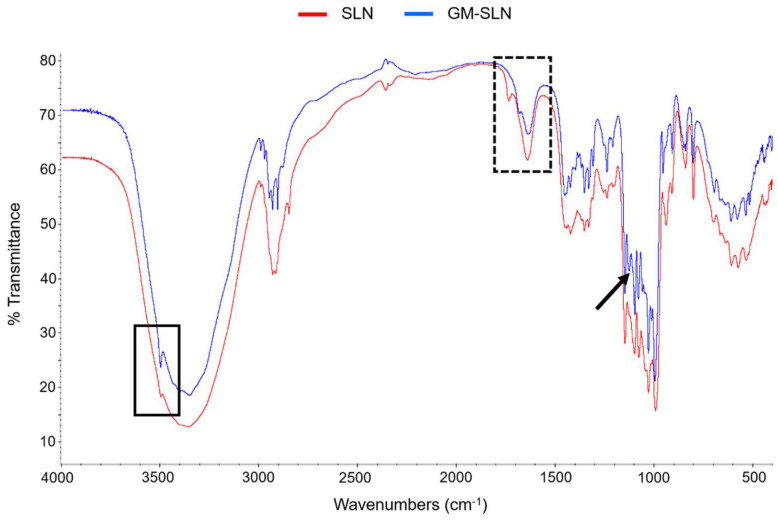
Fourier Transform Infrared Spectroscopy (FT-IR) spectra of SLNs and the GM-SLN vector. Black arrow points to the peak at around 1150 cm^−1^ present in the GM-SLN spectrum. Black square indicates the difference in the intensity of peak at 3500 cm^−1^ between the two spectra. Dashed square shows the displacement of bands at around 1700–1800 cm^−1^, probably indicating formation of hydrogen bonds. GM-SLN: galactomannan SLN-based vector. SLN: solid lipid nanoparticle.

To gain insight into the solid-state composition of SLNs, FT-IR analyses were carried out on lyophilized SLNs and GM-SLN. FT-IR is widely employed in literature to ascertain interactions between chemical compounds of drug formulations [41]. Figure 3 shows the FT-IR absorption spectrum of SLNs and GM-SLN.

Both spectra appeared similar except for a displacement of bands at around 1700–1800 cm^−1^ (dashed square), which may indicate formation of hydrogen bonds, and a peak at around 1150 cm^−1^ present in the GM-SLN spectrum (black arrow), related to GM. Additionally, a peak at 3500 cm^−1^ (black square) appeared more intense in the GM-SLN.

### 3.5. Cryo-Transmission Electron Microscopy (Cryo-TEM) Images

**Figure 4:**
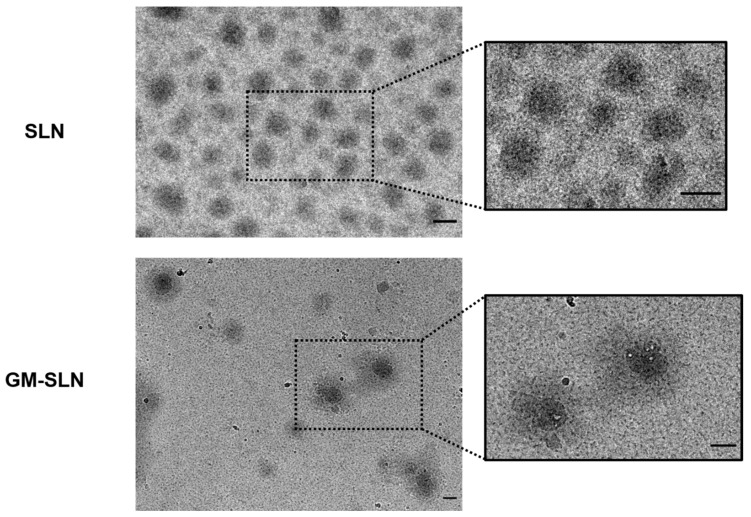
Cryo-Transmission Electron Microscopy (Cryo-TEM) images of the SLNs and the GM-SLN vector. Scale bars = 100 nm. GM-SLN: galactomannan SLN-based vector. SLN: solid lipid nanoparticle.

The morphology of the SLNs and the GM-SLN vector bearing pR-M10-αGal A were analyzed by Cryo-TEM. Figure 4 shows photographs of the SLNs and the GM-SLN vector.

Both, SLNs and vectors, showed a spherical shape and homogeneous distribution. In the case of the GM-SLN vector, a corona can be observed around the SLN that would correspond to the rest of the components of the vector, arranged on the surface of the particles. 

### 3.6. Binding, Protection and Release of the pDNA 

**Figure 5:**
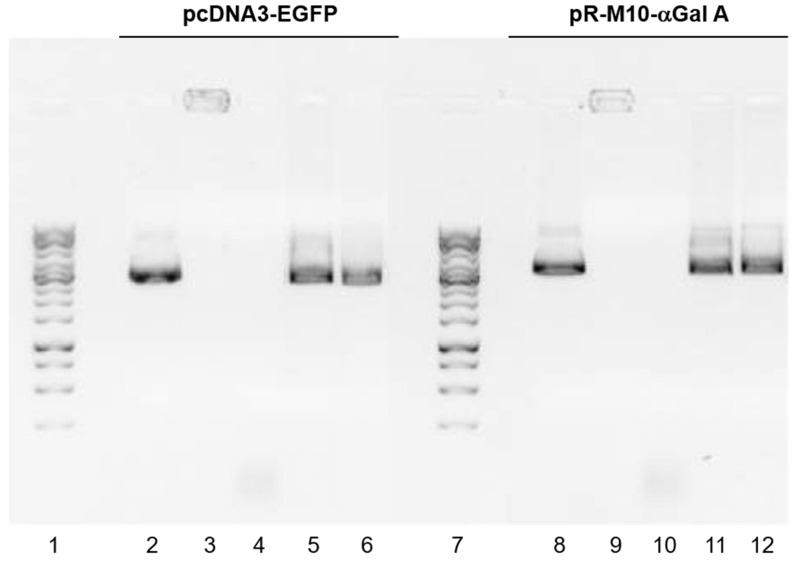
Capacity of the GM-SLN vector to bind, protect and release pcDNA3-EGFP and pR-M10-αGal A by agarose gel electrophoresis. (1) 1 Kb pDNA ladder. (2) Free pcDNA3-EGFP. (3) GM-SLN vector. (4) Free pcDNA3-EGFP + DNase. (5) GM-SLN vector + DNase. (6) GM-SLN vector + SDS. (7) 1 Kb pDNA ladder. (8) Free pR-M10-αGal A. (9) GM-SLN vector. (10) Free pR-M10-αGal A + DNase. (11) GM-SLN vector + DNase. (12) GM-SLN vector + SDS.

The capacity of the GM-SLN vector to bind, protect and release the pDNA was evaluated by agarose gel electrophoresis. Figure 5 shows the ability of the GM-SLN vector to bind, protect and release pcDNA3-EGFP and pR-M10-αGal A pDNAs. The absence of bands in lanes 3 and 9 demonstrates that there is no free pDNA. Additionally, the bands in the sample-loading well of lanes 3 and 9 confirm that both pcDNA3-EGFP and pR-M10-αGal A, respectively, were fully bound and were unable to migrate through the gel. The GM-SLN vector successfully protected both pDNAs from DNase degradation as can be observed in lanes 5 and 11, while free pDNAs were degraded in the presence of DNase (lanes 4 and 10). Regarding the release of the genetic cargo, the vector efficiently released the pDNAs after the treatment with SDS, as it can be seen in lanes 6 and 12.

### 3.7. In Vitro Studies in Hep G2 Cells

#### 3.7.1. Cellular Uptake

**Figure 6:**
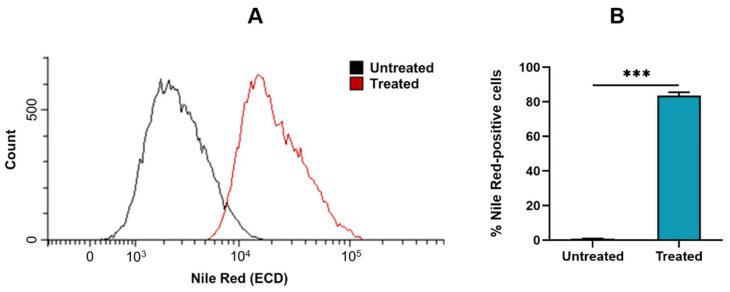
Cellular uptake of the GM-SLN vector by Hep G2 cells. (**A**) Flow cytometry histogram of the cellular uptake study. (**B**) Percentage of Nile Red-positive cells. The percentage of Nile Red-positive cells corresponds to cells containing the Nile Red-labeled GM-SLN vector over total cells. Results are shown as the mean ± standard deviation (*n* = 3). Statistical significance: *** *p* < 0.001.

Cellular uptake of Nile Red-labeled GM-SLN vector by Hep G2 cells was assessed by flow cytometry 2 h after the addition of the vector to cultured cells (Figure 6).

As shown in Figure 6A, the rightward shift of the histogram obtained from the cells treated with the vector demonstrated the efficient internalization of the GM-SLN vector into Hep G2 cells, which was taken up by 83.51 ± 2.02% of cells (Figure 6B).

#### 3.7.2. Intracellular Disposition

**Figure 7:**
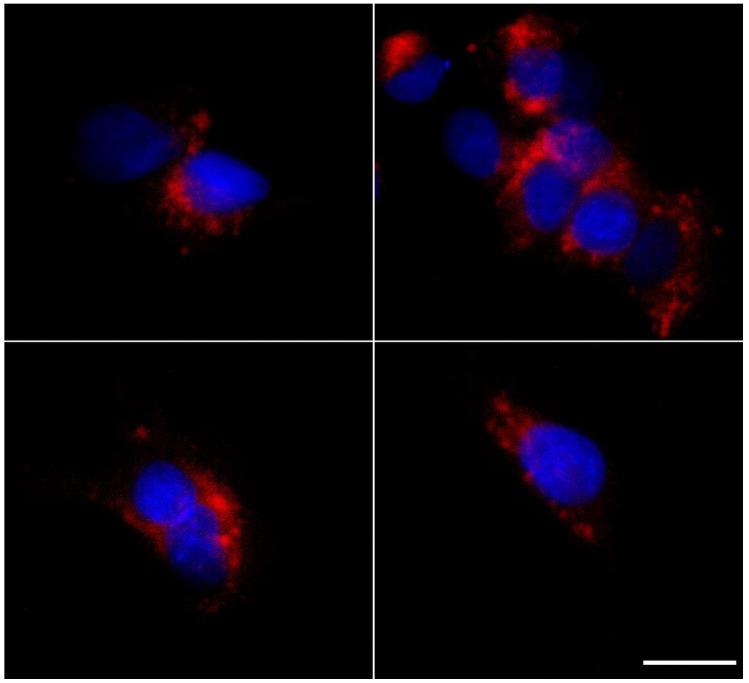
Fluorescence microscopy images of intracellular disposition of the Nile Red-labeled GM-SLN vector 2 h after the addition to Hep G2 cells. Images correspond to different cells of four replicates. Blue: nuclei labeled with DAPI. Red: fluorescence signal of GM-SLN vector prepared with Nile Red-labeled SLNs. Magnification: 60×. Scale bar: 15 µm.

Figure 7 shows the intracellular distribution of Nile Red-labeled GM-SLN vectors after the internalization into Hep G2 cells. The vectors appeared distributed all over the cytoplasm, but mostly in regions close to the cell nucleus.

#### 3.7.3. GFP Transfection Efficacy and Cell Viability

**Figure 8:**
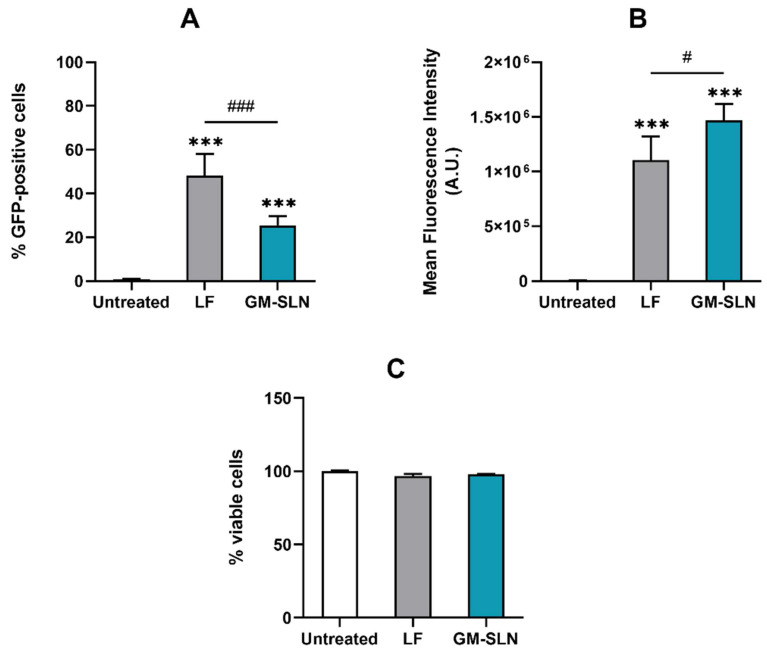
Transfection efficacy and cell viability analysis by flow cytometry in Hep G2 cells 3 days post-transfection. (**A**) Percentage of GFP-positive cells. The percentage of GFP-positive cells corresponds to fluorescent GFP cells over total cells. (**B**) Mean fluorescence intensity. Mean intensity of fluorescence indicates the average intensity of fluorescence per labeled cell. (**C**) Percentage of viable cells. Results are shown as the mean ± standard deviation (*n* = 3). Statistical significance: *** *p* < 0.001 in comparison to untreated cells. ^#^ *p* < 0.05, ^###^ *p* < 0.001. A.U.: arbitrary unit. GFP: green fluorescent protein. GM-SLN: galactomannan SLN-based vector. LF: Lipofectamine^®^ 2000.

Transfection efficacy and cell viability were assayed in Hep G2 cells by flow cytometry 3 days after the addition of the GM-SLN vector bearing the pcDNA3-EGFP pDNA to the cells. Figure 8 represents the percentage of GFP-positive cells (A), the mean fluorescence intensity (B), and cell viability (C). Results were compared with Lipofectamine^®^ 2000 (positive control).

As shown in Figure 8A, the GM-SLN vector was able to transfect around 25% of the cells. Although the percentage of positive cells was lower with the vector than with the positive control Lipofectamine^®^ 2000, the mean fluorescence intensity, which correlates with protein production, was higher with the GM-SLN vector (Figure 8B). Cell viability was similar to that observed in untreated cells and was always close to 100% of viable cells (Figure 8C).

#### 3.7.4. α-Galactosidase A Transfection

**Figure 9:**
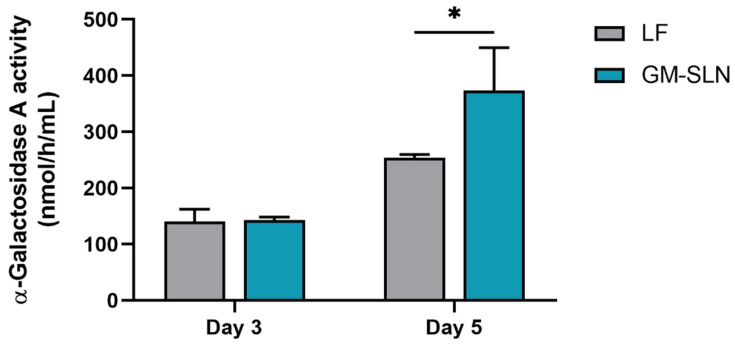
α-Galactosidase A activity in the culture medium of Hep G2 cells, 3 and 5 days post-transfection. One unit of α-Galactosidase A activity is equivalent to the hydrolysis of 1 nmol of the substrate 4-methylumbelliferyl-α-D-galactopyranoside in 1 h at 37 °C. Results are shown as the mean ± standard deviation (*n* = 3). Statistical significance: * *p* < 0.05. GM-SLN: galactomannan SLN-based vector. LF: Lipofectamine^®^ 2000.

To evaluate the capacity of the GM-SLN vector to induce the synthesis of α-Gal A in vitro, α-Gal A activity was quantified in the culture medium of Hep G2. Figure 9 represents α-Gal A activity in the culture medium of cells 3 and 5 days after the transfection with the GM-SLN vector bearing pR-M10-αGal A. Results were compared with Lipofectamine^®^ 2000 (positive control). The basal α-Gal A activity of untreated cells was < 1 nmol/h/mL.

In all cases, α-Gal A activity significantly increased with respect to basal α-Gal A activity of untreated cells (<1 nmol/h/mL). On day 3, the enzyme activity was equal to that obtained with the positive control Lipofectamine^®^ 2000. Five days after the addition of the GM-SLN vector, the α-Gal A activity in Hep G2 cells increased to 373 nmol/h/mL, being 1.5 times higher than the commercial transfection reagent Lipofectamine^®^ 2000. 

### 3.8. Interaction with Erythrocytes: Hemolysis and Hemagglutination

**Figure 10:**
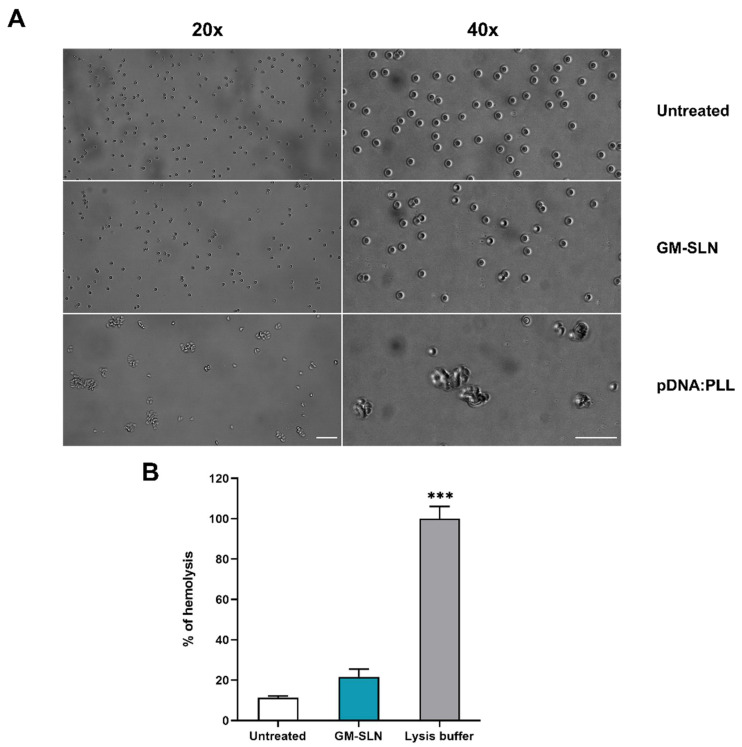
Interaction of GM-SLN with erythrocytes. (**A**) Agglutination of erythrocytes. (**B**) Hemolytic activity. Lysis buffer represents 100% hemolysis sample. Results are shown as the mean ± standard deviation (*n* = 3). Scale bars = 50 µm. Statistical significance: *** *p* < 0.001. GM-SLN: galactomannan SLN-based vector. pDNA: plasmid DNA. PLL: poly-L-lysine.

Hemagglutination was evaluated by incubating the GM-SLN vector with erythrocytes. The photographs in Figure 10A show no agglutination of erythrocytes with the GM-SLN vector.

Figure 10B features the hemolytic activity. As can be seen, the GM-SLN vector did not show hemolysis of erythrocytes.

### 3.9. In Vivo Intravenous Administration of the GM-SLN Vector to α-Gal A KO Mice

**Figure 11:**
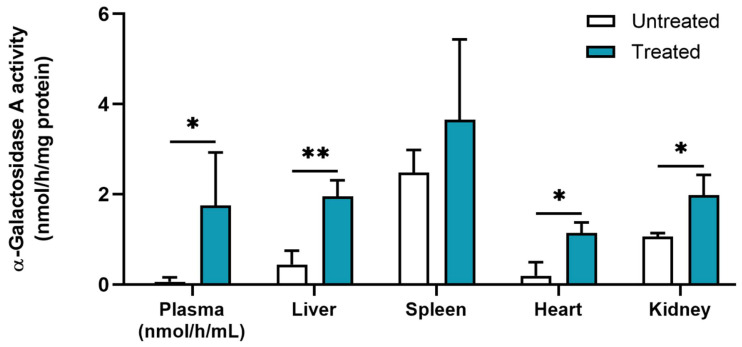
α-Galactosidase A activity in plasma and tissues of untreated and treated mice 5 days after the intravenous administration of the GM-SLN vector. One unit of α-Galactosidase A activity is equivalent to the hydrolysis of 1 nmol of the substrate 4-methylumbelliferyl-α-D-galactopyranoside in 1 h at 37 °C. Results are shown as the mean ± standard deviation (*n* = 3). Statistical significance: * *p* < 0.05, ** *p* < 0.01.

The efficacy of the GM-SLN vector to increase α-Gal A activity in vivo was evaluated in α-Gal A KO mice. Figure 11 represents the α-Gal A activity in plasma, liver, spleen, heart and kidney of untreated and treated mice, five days after the i.v. administration of the GM-SLN vector.

Enzyme activity significantly increased in plasma, liver, heart and kidney of treated mice with respect to the untreated group. The greatest difference was observed in plasma. In the liver, α-Gal A activity was four times higher than in untreated animals. Additionally, the administration of the GM-SLN vector resulted in a six- and two-fold increase in heart and kidney, respectively. No significant difference was seen in the spleen. 

Results of enzyme activity in treated animals were also evaluated in means of the percentage of activity achieved regarding the levels in wild-type mice [32] (Table 2).

As it is shown in Table 2, after the treatment with the GM-SLN, α-Gal A activity reached 18% of the enzyme activity of wild-type mice in plasma, 28% in heart and 14% in kidney. 

## 4. Discussion

Composition and preparation method are two key considerations in the design and production of non-viral systems. Here, the vector was prepared with pDNA, protamine (P), galactomannan (GM) and SLNs. To prepare the vector, first pDNA (either pcDNA3-EGFP or pR-M10-αGal A) was mixed with P, followed by the addition of GM. SLNs were added to GM-P-pDNA complexes, leading to the adsorption of the complexes on the surface of the SLNs by electrostatic interactions to obtain the GM-P-pDNA-SLN vector. P is a polycationic peptide that has been extensively employed to enhance lipofection. Due to its cationic nature, P binds the pDNA and protects it from degradation. Moreover, P has nuclear localization signals in its sequence which promote the entry of the genetic material into the nucleus of cells and, therefore, it represents a great carrier for pDNA [42,43]. GMs are biodegradable heterogeneous polysaccharides composed by a β-(1–4)-D-mannan backbone with a single D-galactose branch linked α-(1–6) [44]. The role of carbohydrate ligands in enabling asialoglycoprotein (ASGPR)-mediated targeting is broadly demonstrated. The ASGPR receptor is expressed mainly on hepatocytes and minimally on extra-hepatic cells, and exhibits a high affinity for D-galactose end groups [45]. Therefore, the addition of GM to the vector is expected to favor the interaction and entry into hepatic cells after systemic administration, with the aim of targeting the liver as an α-Gal A production organ [13,14,17,18,19,20,21].

The SLNs prepared in the present work are a type of LNs made of a core of solid lipids at room temperature, dispersed in an aqueous solution stabilized by surfactants, which usually includes cationic lipids that allow electrostatic interactions with nucleic acids [46,47]. SLNs were prepared by a hot-melt emulsification technique, which is low-cost, easy, and reproducible. Importantly, it avoids the use of organic solvents. The process involves using ultrasonication, which has been widely used for the preparation of different nanomaterial-based particles [48,49,50], including LNs [47]. Increasing sonication time reduced particle size of SLNs prepared with 100 or 200 mg of Precirol^®^ ATO 5 (Figure 1A). Parameters for the synthesis of SLNs were set at 100 mg of Precirol^®^ ATO 5 and 30 min sonication, since with those conditions the particle size was below 100 nm. In addition, SLNs did not show changes in physicochemical characteristics (particle size, polydispersity index and ζ-potential) during five weeks at 4 °C (Figure 2), indicative of good colloidal stability. The prepared SLN-based vectors showed a size suitable for i.v. administration, 97.3 ± 2.8 nm and low polydispersity index (0.17 ± 0.02). The GM-SLN vector presented a ζ-potential (+33.6 ± 1.6 mV) significantly lower than SLNs alone. The decrease in charge is the result of the adsorbtion of GM-P-pDNA complexes on the surface of the SLN, which mask part of the positive charges on the surface. The change in surface charge together with the change in FT-IR spectra (Figure 3) confirm the presence of the GM-P-DNA complex on the SLN surface. In addition, the corona observed around the SLN in cryo-TEM images of GM-SLN vector (Figure 4) is indicative of the presence of these complexes arranged on the surface of the nanoparticles. Nonetheless, the final vector maintains the cationic nature, which is beneficial for transfection efficiency, since it is well known that cationic delivery systems electrostatically interact with the negatively charged glycoproteins and proteoglycans of the cell membrane and may facilitate cellular uptake [46,51,52,53,54]. In fact, the vector showed a high internalization capacity (>80%) in vitro into liver-derived cells, Hep G2 (Figure 6), and intracellular disposition studies revealed localization of the vector within the cell in regions close to the nucleus (Figure 7). This cell line has been used as a model for the evaluation of gene therapy strategies for FD by other authors [19].

Transfection mediated by non-viral vectors also depends largely on the balance between the capacity of the system to bind the nucleic acid and protect it from degradation, and the ability to release it into the intracellular compartment. In systemic gene delivery, degradation by nucleases is considered one of the most compromising limitations [23,55]. The GM-SLN vector was able to bind and release the pDNA, and to efficiently protect it from DNase degradation (Figure 5).

To demonstrate the transfection ability, the GM-SLN vector was firstly prepared with a pDNA encoding GFP (pcDNA3-EGFP), which allows knowing the percentage of transfected Hep G2 cells. In comparison with Lipofectamine^®^ 2000, one of the most commonly used commercial reagents for in vitro delivery of nucleic acids [56], the GM-SLN vector transfected a lower percentage of Hep G2 cells, (Figure 8A), but the mean intensity of fluorescence, which correlates with the amount of protein produced [38,39], was significantly higher (Figure 8B). The delivery system highly influences the internalization pathway and intracellular disposition of the genetic material. These processes condition the access to the cellular machinery necessary to produce the corresponding protein, and, therefore, the transfection efficacy of non-viral vectors [43]. Considering this, results indicate that the GM-SLN vector is more efficient than Lipofectamine^®^ 2000 in promoting protein synthesis in transfected cells. In vitro transfection studies with the pDNA encoding α-Gal A (pR-M10-αGal A) confirmed this, since the enzyme activity 5 days after transfection was higher with the GM-SLN vector than with Lipofectamine^®^ 2000 (Figure 9). Moreover, measuring the α-Gal A activity was useful to ensure that the expressed enzyme was functional.

Considering the systemic administration, before in vivo studies, we confirmed that the vector did not show relevant agglutination of erythrocytes (Figure 10A), and that the formulation lacked hemolytic activity (Figure 10B). This is an advantage over other cationic transfection agents, such as poly-L-lysine (PLL) or polyethylenimine (PEI)-based systems. PLL and PEI have been extensively used as nucleic acid delivery systems owing to their high charge density, which is essential for effective nucleic acid complexation [57,58]. However, it is well known that positive charges of cationic systems may interact with blood components after systemic administration, such as serum proteins and blood cells. The interaction leads to aggregation and hemagglutination and, consequently, to high clearance from the blood stream by the reticuloendothelial system [59,60,61]. 

Finally, the GM-SLN vector was administered to α-Gal A KO mice, an animal model of FD, to assess its capacity to increase in vivo enzyme activity levels. A single i.v. administration to mice produced a significant increase of α-Gal A activity in plasma, liver, heart and kidney with respect to the untreated group (Figure 11). Circulating α-Gal A in plasma reveals that the tissues transfected by the GM-SLN vector were capable of producing and releasing the enzyme in sufficient quantity to be secreted, which is the basis for an efficient cross-correction. In a previous study, we evaluated in the same animal model a non-viral vector functionalized with dextran (DX-SLN) with a larger particle size (233 ± 10.5 nm) than the GM-SLN vector [32]. The administration of a single i.v. dose of the DX-SLN vector to mice resulted in increased α-Gal A activity respect to non-treated mice only in spleen and kidney, and three doses, one per week, were needed to increase enzyme activity in liver and heart. In the present work, one dose of GM-SLN was enough to achieve enzyme activity levels in the liver similar to those previously obtained with a multiple-dosage regimen of DX-SLN. In addition, plasma levels were at least six times higher than those reported in the previous work after single or multiple dosing of DX-SLN. The higher plasma levels with GM-SLN may be related both, to the expression in liver, heart and kidney, and to a reduced clearance of the vector by the spleen, where we did not find differences in α-Gal A activity with respect to non-treated mice. Accumulation of nanoparticles in the spleen is mainly attributed to those larger than 200 nm that can activate the complement system and be taken up by reticuloendothelial system cells [62]. 

The heart and kidneys are two of the most damaged organs in FD, and affectation of these tissues is the major cause of morbidity and mortality in Fabry patients [2,63,64]. Therefore, although the GM-SLN vector was designed to enhance liver affinity, off-targeting to these organs is not a problem for this kind of multi-organ diseases. In fact, one of the objectives of therapies for FD is to reduce Gb3 deposits mainly in heart and kidneys [4,6,65]. The GM-SLN formulation was able to increase enzyme activity in these organs to 28% and 14% of activity of the wild-type, respectively. These results are of special relevance since it has been shown that 10% of the wild-type enzyme activity is enough to sufficiently clear deposits of Gb3 in various organs [16,66,67]. 

## 5. Conclusions

In this study, the hot-melt emulsification technique was useful to obtain SLN-based non-viral vectors below 100 nm in size for gene supplementation in FD. The vector functionalized with GM, a polysaccharide containing galactose groups, was highly efficient in promoting protein synthesis in a liver cell line in vitro, and clinically relevant α-Gal A activity levels were achieved in different organs after systemic administration to an animal model of FD. The levels of activity attained in the heart and kidneys are noteworthy, because these are two of the most damaged organs in FD. Both the reduced size of the vector and the functionalization with GM influence the in vivo transfection efficiency of the vector and the biodistribution of the enzyme, offering a novel approach for the treatment of FD by pDNA-based gene augmentation. 

## Figures and Tables

**Table 1:** Characterization of the GM-SLN vector. Z-Average, polydispersity index and ζ-Potential of the GM-SLN vector bearing pcDNA3-EGFP or pR-M10-αGal A.

	Z-Average (d.nm)	PDI	ζ-Potential (mV)
pcDNA3-EGFP			
GM-SLN	98.3 ± 0.9	0.20 ± 0.02	+35.2 ± 1.4
pR-M10-αGal A			
GM-SLN	97.3 ± 2.8	0.17 ± 0.02	+33.6 ± 1.6

GM-SLN: galactomannan SLN-based vector; PDI: polydispersity index; SLN: solid lipid nanoparticle. Results are shown as the mean ± standard deviation (*n* = 3).

**Table 2:** Percentage of α-Galactosidase A activity of wild-type mice in treated mice with the GM-SLN vector.

**% of Wild-Type**	**Plasma**	**Liver**	**Spleen**	**Heart**	**Kidney**
	18	6	7	28	14
